# Self-Reported Late Effects, Information Needs, and Preferences for Long-Term Follow-Up Care Among Survivors of Childhood Cancer: A Nationwide Survivor-Led Survey from Germany

**DOI:** 10.3390/cancers18182951

**Published:** 2026-09-12

**Authors:** Jette Luedersen, Bjoern Hessing, Marie Alfes, Franziska E. Marquard, Eva-Maria Wild

**Affiliations:** 1 Survivor Deutschland e.V., Deutscher Kinderkrebsverband, 53175 Bonn, Germany; j.luedersen@uke.de (J.L.); bjoern.hessing@nct-heidelberg.de (B.H.); marie.alfes@abbvie.com (M.A.); 2 Department of Stem Cell Transplantation, University Medical Center Hamburg-Eppendorf, 20246 Hamburg, Germany; f.marquard@uke.de; 3 Exercise Oncology Research Group, Department of Medical Oncology, Heidelberg University Hospital, Medical Faculty Heidelberg, Heidelberg University, 69120 Heidelberg, Germany; 4 National Center for Tumor Diseases (NCT), 69120 Heidelberg, Germany; 5 Parenteral Product Development Science and Technology Analytical, AbbVie Deutschland GmbH & Co. KG, 67061 Ludwigshafen, Germany; 6 Department of Paediatrics and Adolescent Medicine, University Hospital Erlangen, Friedrich-Alexander-Universität Erlangen-Nürnberg (FAU), 91054 Erlangen, Germany; 7 Pediatric Oncology Network Bavaria, Kinderonkologisches Netzwerk (KioNet), 91054 Erlangen, Germany; 8 Bavarian Cancer Research Center (BZKF), 91052 Erlangen, Germany

**Keywords:** childhood cancer survivors, late effects, daily-life impairment, survivorship care, long-term follow-up, patient preferences, unmet needs, survivor involvement, psychosocial care, Germany

## Abstract

**Background**: Rising survival rates have created a growing population of childhood cancer survivors (CCSs) who have an increased risk of late effects and require long-term follow-up (LTFU) care. Existing services are often fragmented and may not reflect survivors’ priorities; optimizing such care requires understanding not only the self-reported burden of late effects and their impact on daily lives but also survivors’ information status and their preferences for future care. **Methods**: Survivor Deutschland e.V. conducted a nationwide cross-sectional online survey assessing the current late effects, daily-life impairments, subjective information statuses, current follow-up structures, and preferences for future LTFU care. A total of 339 CCSs were included, covering all childhood cancer entities, most frequently leukemia (30.7%), central nervous system tumors (18.3%), and lymphoma (16.5%). Data were analyzed descriptively and supplemented by paired non-parametric analyses. **Results**: Overall, 74.3% (252/339) reported at least one late effect. Self-rated limitations in daily life had a median of five (Q1–Q3 3–7) on a 1–10 scale, which increased with the number of reported late effects. The most frequently affected domains were endocrine (36.3%, *n* = 123), fertility (33.6%), psychological (30.4%, *n* = 103), neurocognitive (26.0%, *n* = 88), and orthopedic (24.8%, *n* = 84) problems. A majority (69.3%, *n* = 235) knew that late effects existed yet felt insufficiently informed, and 4.4% only learned of these through the survey. Among 172 survivors in adult follow-up care, only 26.8% (46/172) reported access to structured, specialized LTFU care, whereas 56.4% (97/172) preferred this model. Survivors rated the importance of LTFU care highly (median 9/10) but rated satisfaction with their current care as much lower (median 3/10). The most valued components were the coverage of follow-up costs, sufficient consultation time, a dedicated contact person, and clear communication of results. Psychological support was a notable gap (5.8%, 10/172 current access vs. 17.9% 31/172 preferred), and 84.1% were willing to travel up to two hours or more for high-quality care. **Conclusions**: German CCSs report a high late-effect burden, meaningful impairment of their daily lives, a pronounced information gap, and substantial unmet care needs. These patient-centered findings support structured, risk-adapted LTFU with proactive information, integrated psychological support, and sustainable financing at specialized LTFU centers.

## Simple Summary

Childhood cancer survivors have an increased risk of late effects and require long-term follow-up care. In a nationwide survey run by survivors themselves, 339 adult survivors of childhood cancer in Germany reported which late effects they currently experience, how much these limit their everyday lives, how well-informed they feel about late effects, what follow-up care they currently receive, and what care they would prefer in the future. Almost three in four reported at least one late effect, and everyday limitation was rated in the middle-to-upper range. Most survivors knew that late effects existed but felt insufficiently informed; a few even heard about them for the first time in the survey. Only about one in four survivors in adult follow-up care had access to structured, specialized care, whereas most would prefer a specialized center and would travel two hours or more for high-quality care. Psychological support was a major unmet need. Our findings support the development of structured, multidisciplinary long-term follow-up centers, with proactive information and sustainable financing.

## 1. Introduction

Survival after childhood cancer now exceeds 80% in high-income countries, with approximately 41,000 childhood cancer survivors (CCSs) in Germany [1,2]. Improved survival has shifted the burden from mortality to long-term morbidity, as many CCSs develop physical or psychological late effects that can emerge years after therapy [3,4]. The St. Jude Lifetime Cohort demonstrated that chronic conditions accumulate throughout adulthood and exceed those in the general population [3]. However, evidence has been largely derived from examination-based cohorts using direct assessment and medical records [3,5,6], which do not capture survivors’ lived experiences, information statuses, or care preferences.

Long-term follow-up (LTFU) care provides structured, risk-adapted, multidisciplinary survivorship care and management of late effects [7]. International initiatives provide risk-based surveillance recommendations [7,8], while the recently published German S2k guideline emphasizes multidisciplinary care, structured transitions to adult care, psychological support, and health promotion [9]. In practice, only 14 LTFU clinics existed in Germany when the survey was conducted [10] and follow-up structures were not yet standardized, while existing services remain fragmented. Whether they reflect survivors’ priorities is largely unknown.

Here, we report a nationwide, survivor-led survey conducted by Survivor Deutschland e.V. that assesses self-reported late effects, daily-life impact, information status, current LTFU care, and future care preferences. This survivor perspective complements clinical cohorts and is internationally relevant given persistent disparities in information provision and LTFU care.

## 2. Materials and Methods

### 2.1. Study Design and Participants

**Figure 1:**
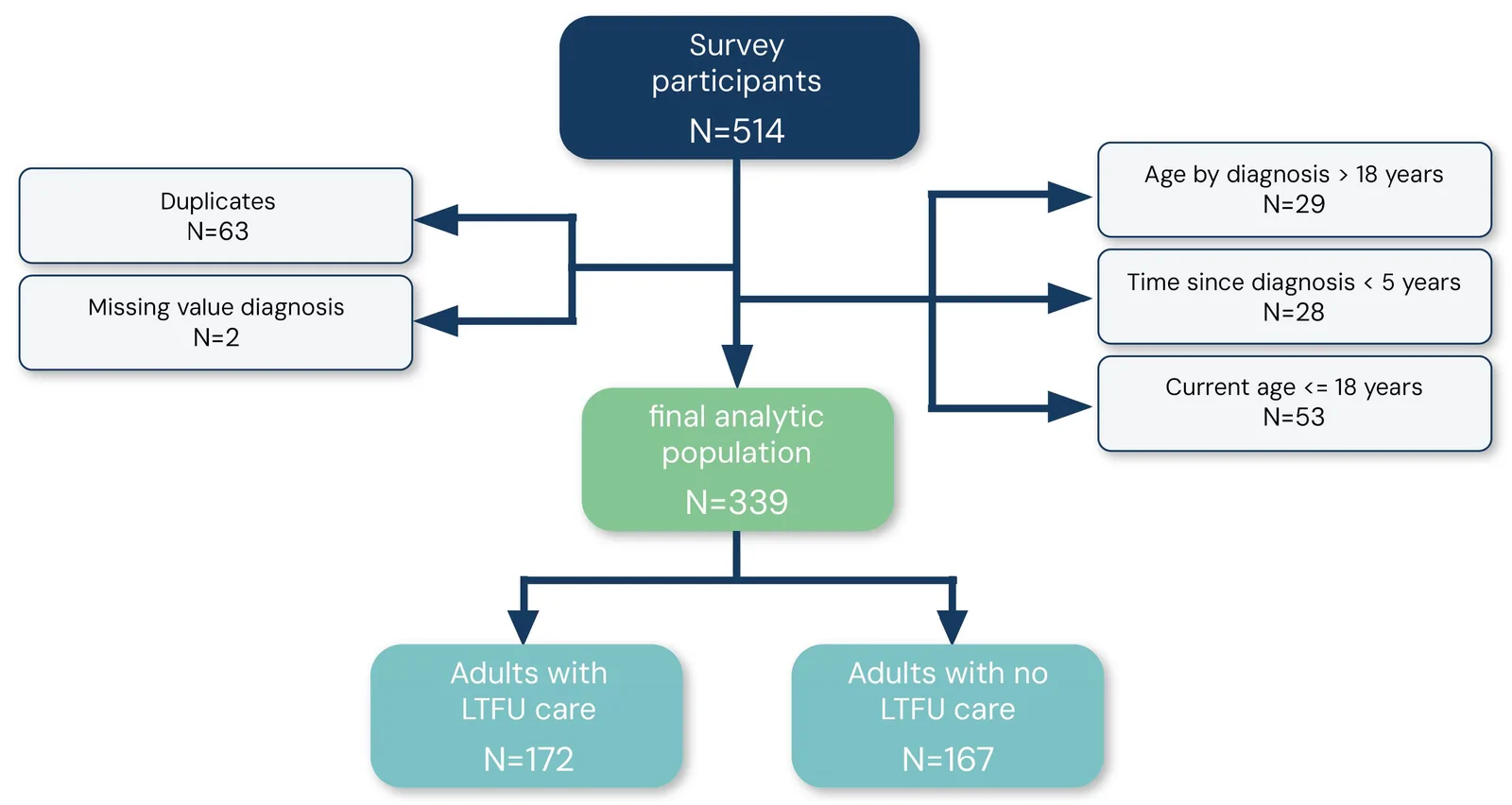
Participant flow chart. A total of 175 survey records were excluded from the original survey sample (n = 514) following data cleaning and the application of exclusion criteria.

Survivor Deutschland e.V. conducted a nationwide, survivor-led cross-sectional online survey from May 2025 to May 2026, recruiting through Survivor Deutschland e.V., collaborating hospitals, patient organizations, survivorship networks, and social media. A total of 514 survivors participated. Cancer survivorship [11] begins at diagnosis and extends throughout the remainder of life, including individuals in remission, undergoing active treatment, and those living with chronic or advanced cancer. CCSs were defined as individuals diagnosed with cancer before 18 years. Inclusion criteria comprised CCSs who were aged ≥18 years at the time of participation and >5 years from their initial diagnosis, consistent with the commonly used definition of long-term childhood cancer survivorship [12], as the risk of recurrence decreases substantially over time [13,14]. After excluding incomplete, duplicate, and non-eligible records, 339 CCSs formed the analytical cohort. A detailed participant flow diagram and dropout table is shown in Figure 1.

### 2.2. Survey Instrument and Measures

The questionnaire was developed through a survivor-led co-design with clinicians, researchers, and patient input. It was pilot tested for comprehensibility and feasibility. The questionnaire assessed cancer and treatment history, late effects, daily-life impairment, information status, current access to and satisfaction with LTFU care, and future care preferences. LTFU was defined as lifelong, risk-adapted surveillance that aims to prevent, detect, and manage treatment-related late effects [9,15]. Structural components of survivorship care were rated on a 10-point Likert scale. Primary-analysis questions were mandatory; conditional questions followed previous responses. Multiple responses were allowed where appropriate. The full questionnaire is provided in Supplementary Material S1.

### 2.3. Statistical Analysis

All analyses were performed in Python 3.13 using pandas 2.3.3, NumPy 2.3.5, SciPy 1.16.3, and Matplotlib 3.10.7. Categorical variables are reported as frequencies and percentages, using Wilson 95% confidence intervals for key proportions and ordinal variables as medians (IQR, Q1–Q3), supplemented by means and standard deviations where appropriate. The association between the number of self-reported late effects and daily-life impairment was assessed using Spearman’s correlation; group differences using Kruskal–Wallis or Mann–Whitney U tests. Category-specific impairment estimates were interpreted descriptively because multiple late effects could be reported. Information status was summarized overall and by age at diagnosis, diagnosis period, and time since diagnosis; subgroup analyses were exploratory.

Paired ratings of LTFU care and satisfaction were compared using the Wilcoxon signed-rank test with rank-biserial correlation. Current versus preferred access to psychological support was paired and analyzed using the exact McNemar test with paired odds ratios (complete pairs, n = 172). Tests were two-sided, with *p* < 0.05 considered to be statistically significant. Self-reported late-effect proportions were descriptively contextualized with examination-based cohorts but not formally compared because of methodological and population differences. Analyses and figures were generated using reproductible scripts.

### 2.4. Ethics

The study used fully anonymized survey data without personal identifiers. The Ethics Committee of Friedrich-Alexander-Universität Erlangen-Nürnberg confirmed that, pursuant to Section 15 of the Bavarian Professional Code of Conduct for Physicians, ethics approval was not required for research using fully anonymized data (reference number: 25-374-ANF; 8 September 2025). Participation was voluntary, and electronic informed consent was obtained from all participants before their inclusion in the study.

## 3. Results

### 3.1. Cohort Characteristics

Of the 339 eligible respondents, all provided complete demographic and clinical data. The median age was 31 years (Q1–Q3 25–38), the median age at diagnosis was 10 years (Q1–Q3 4–14), and the median time since initial diagnosis was 22 years (Q1–Q3 16.5–30). Respondents were predominantly female (67.0%, n = 227), while 32.2% (n = 109) were male and 0.9% (n = 3) were gender diverse (see Table 1).

The most common primary diagnoses were leukemia (30.7%, n = 104), central nervous system tumors (18.3%, n = 62), lymphoma (16.5%, n = 56), bone tumors (12.4%, n = 42), and soft tissue sarcoma (8.0%, n = 27). Years of diagnoses spanned 1970 to 2020 and peaked in 2000–2009 (134/339). Eighty-five CCSs (25.1%) had experienced at least one relapse. Most survivors had received chemotherapy (90.6%, n = 307) or surgery (55.5%, n = 188); radiotherapy was reported separately as cranial irradiation (27.4%, n = 93), local irradiation (26.0%, n = 88), and total body irradiation (12.1%, n = 41). A total of 67 (19.8%) received stem cell transplantation.

### 3.2. Self-Reported Burden and Daily-Life Limitation

Overall, 74.3% (n = 252) of CCSs reported at least one late effect. The most frequently reported domains were endocrine (36.3%), fertility (33.6%), psychological (30.4%), neurocognitive (26.0%), and orthopedic (24.8%) problems. Fertility problems were reported by 34.4% of female and 31.2% of male respondents.

**Figure 2:**
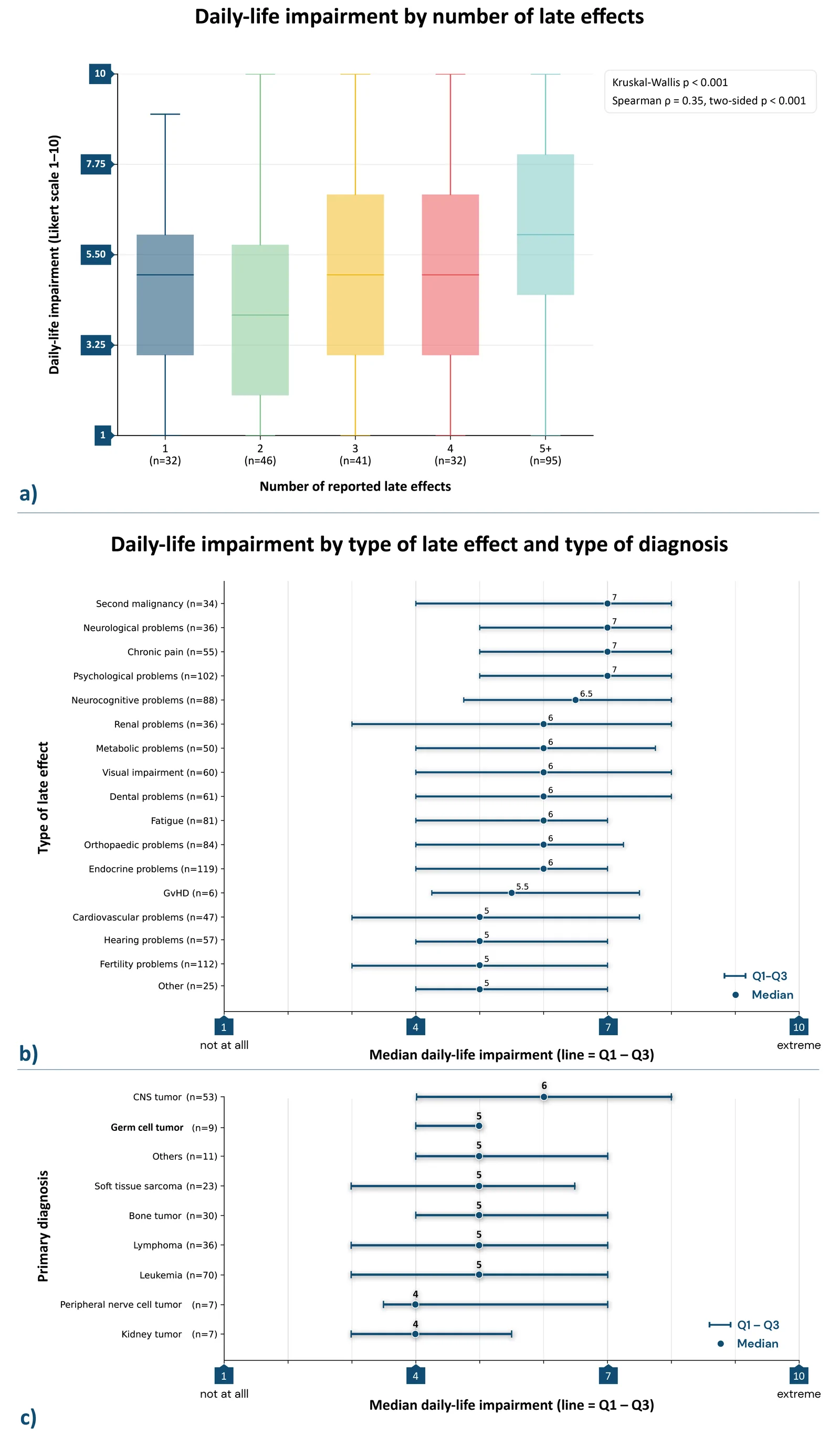
(**a**) Daily-life limitation (1–10 Likert) of CCSs by number of reported late effects (box plots; box = Q1–Q3, horizontal line = median). (**b**) Median daily-life limitation (1–10 Likert) of CCSs by type of late effects, with n per category (error bars = Q1–Q3). (**c**) Daily-life limitation (1–10 Likert) of CCSs by primary diagnosis (median; error bars = Q1–Q3).

Self-rated limitation in daily life had a median of five (Q1–Q3 3–7) on a 1–10 scale, which increased with the number of reported late effects (Spearman’s ρ = 0.35, two-sided *p* < 0.001), rising to a median of about six among survivors reporting five or more late effects (Figure 2a). It also varied by the type of late effect (Figure 2b), as the median limitation was highest for second malignancy, neurological, chronic-pain, and psychological problems (all median seven) and lower for fertility, hearing, and cardiac problems (median five). By primary diagnosis, survivors of CNS tumors reported the numerically highest median impairment (median six, Q1–Q3 4–8), although the overall difference across diagnostic groups was not statistically significant (Kruskal–Wallis *p* = 0.850; Figure 2c); impairment also did not differ significantly between female and male survivors (both median five; Mann–Whitney *p* = 0.069).

### 3.3. Information Status

Overall, 69.3% of CCSs (n = 235) knew that therapy can cause late effects but felt insufficiently informed, whereas 26.3% (n = 89) felt adequately informed and 4.4% (n = 15) first learned about late effects through the survey. This information gap was consistent across subgroups: insufficient information predominated across all strata of age at diagnosis, diagnosis period, and time since diagnosis (see Supplementary Figure S1a–c). Descriptively, sufficient information was reported more often by survivors diagnosed during adolescence (35–46% across the displayed categories) than by those diagnosed at younger ages (18–27%), whereas neither the diagnosis era nor time since diagnosis showed a consistent trend. Feeling sufficiently informed did not increase with longer follow-up. Insufficient information predominated across all diagnostic groups and was more common among female survivors (about 22% versus 37%). Information status also differed by the current type of adult follow-up care; no CCS attending specialized LTFU care first learned about late effects through the survey (see Supplementary Figure S2).

### 3.4. Current Long-Term Follow-Up Care

Overall, 50.7% of participants (n = 172) reported currently receiving ongoing or reinitiated adult follow-up care, whereas 35.1% (n = 119) had discontinued their LTFU care upon transition to adult care at 18 years of age or for other reasons, and 10.9% (n = 37) reported never having received any form of LTFU care. Stated reasons for not receiving LTFU care were primarily insufficient information (24.2%, n = 82), limited access (19.5%, n = 66), or physicians considering it unnecessary (18.0%, n = 61); few avoided follow-up to not be reminded of their cancer history (3.2%, n = 11).

Before the age of 18 years, most CCSs received follow-up care at their pediatric oncology clinic (44.8%, n = 152). Of 172 in current or reinitiated adult follow-up care, most attended community care specialists (44.8%, n = 77), followed by pediatric oncology (15.1%, n = 26) and general practitioners (13.4% (=23), while only 26.8% (n = 46) accessed specialized LTFU care.

### 3.5. Importance of and Satisfaction with LTFU Care

Survivors rated the importance of LTFU care highly (median 9/10, Q1–Q3 7–10), whereas satisfaction with their current care was substantially lower (median 3/10, Q1–Q3 1–8). The median within-person difference was six points, and the paired ratings differed significantly (Wilcoxon signed-rank test: W = 964, two-sided *p* < 0.001; r_r_^b^ = 0.95). As importance and satisfaction represent distinct constructs, this difference indicates a pronounced perceived mismatch but should not be interpreted as a validated gap score or a direct measure of care quality (see Supplementary Figure S3).

### 3.6. Preferences for Future LTFU Care

**Figure 3:**
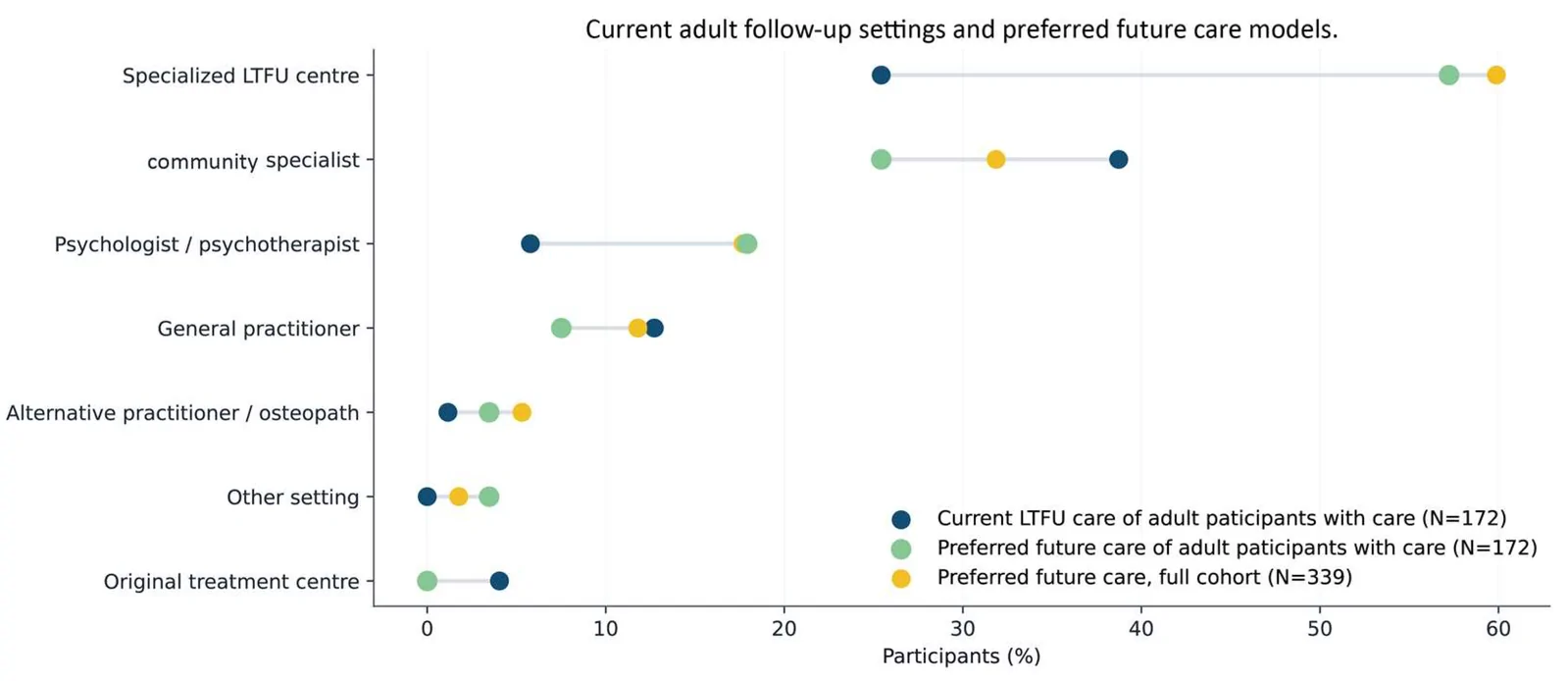
Reported current adult follow-up settings among participants receiving care (*n* = 172), compared with preferred future care settings in the same subgroup and in the total cohort (*n* = 339). Multiple selections were permitted; categories are not mutually exclusive.

In contrast, 59.9% (n = 203) preferred LTFU care in a multidisciplinary center, followed by community specialist (31.9%, n = 108), psychological/psychotherapeutic (17.7, n = 60), and general practitioner care (11.8%, n = 40; see Figure 3).

**Figure 4:**
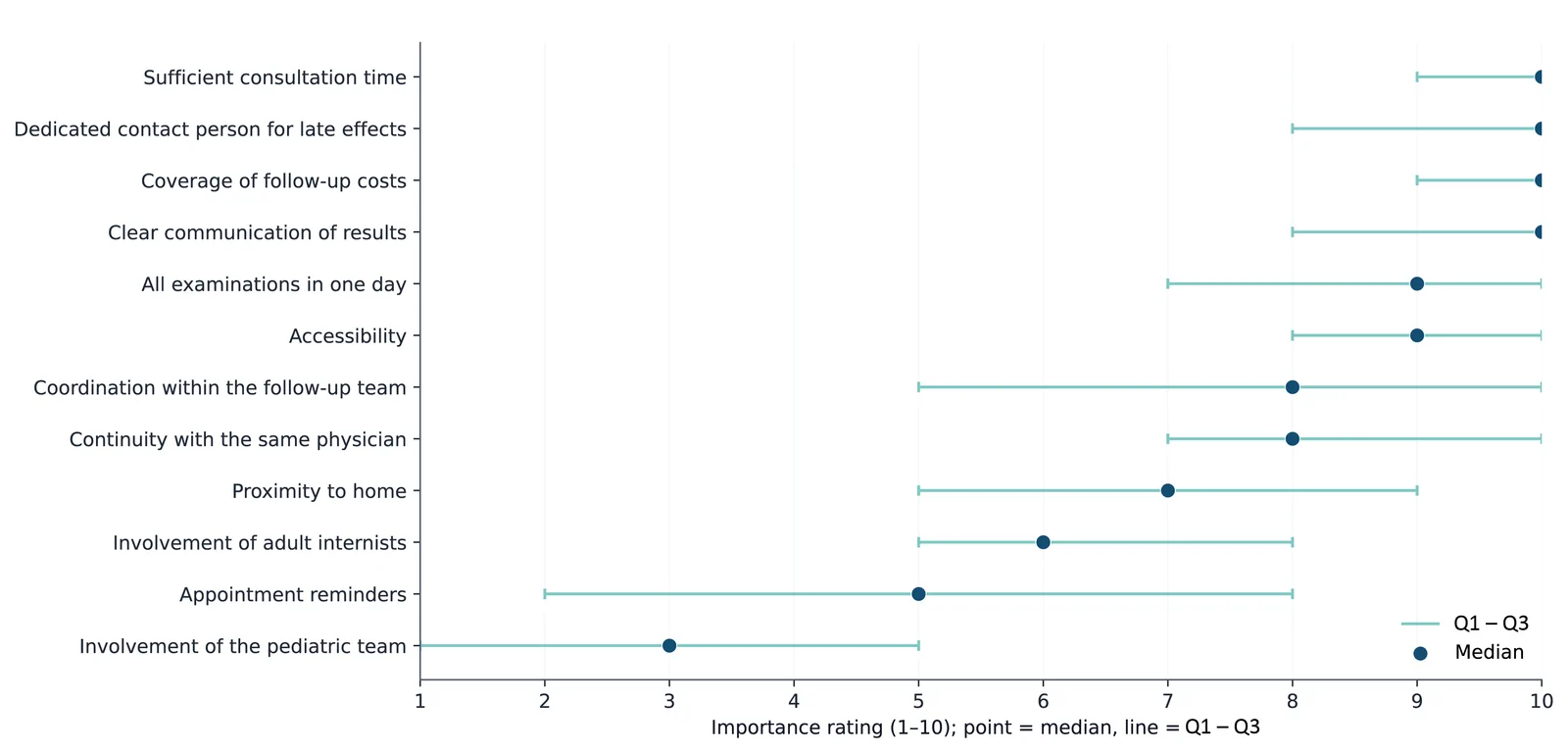
Survivors’ priorities for the organization of long-term follow-up care. Points indicate medians; horizontal lines indicate Q1–Q3.

Among the organizational components of survivorship care, rated on a 10-point scale, the most valued were the coverage of follow-up costs (median 9/10, Q1–Q3 9–10) and sufficient consultation time (median 9/10, Q1–Q3 9–10); a dedicated contact person for late effects, clear communication of examination results, and easy accessibility of specialized services were also rated highly (all median 8/10, Q1–Q3 8–10; see Figure 4).

Notably, most CCSs (84.1%, *n* = 285) were willing to travel up to two hours or more for high-quality LTFU care (Supplementary Figure S4).

Psychological support represented a further gap, as 5.8% (*n* = 10) reported this in their current care, while 17.9% (*n* = 31) preferred its’ inclusion in future LTFU care. Paired analysis showed 28 participants preferred but lacked support versus seven receiving but not preferring it (exact McNemar test *p* < 0.001; paired OR = 4.0).

## 4. Discussion

This nationwide, survivor-led survey of 339 German CCSs revealed a high burden of late effects and consecutive daily-life impairment; persistent information gaps, fragmented follow-up, with limited access to structured, specialized LTFU care; and a strong preference for coordinated, multidisciplinary care. These findings closely align with the German S2k guidelines [9], which promotes patient-centered support within its recommendations.

### 4.1. Cohort Composition and Generalizability

With 339 eligible participants, this is one of the largest survivor-reported surveys on late effects and LTFU care in Germany. Respondents broadly resemble the German long-term survivor population in terms of age, time since diagnosis, and diagnostic distribution [2], with sufficient follow-up for late effects to emerge. The 25.1% relapse proportion, although above current estimates (~12–18%), is plausible for the predominantly 2000–2009 treated cohort and consistent with contemporary international estimates [16]. Recruitment through survivor networks, clinics, and self-help organizations may have selected more engaged survivors and should be considered when interpreting the findings.

### 4.2. Late-Effect Burden and Daily-Life Impairment

Self-rated daily-life impairment increased with the number of reported late effects, whereas no association was observed with sex (*p* = 0.069) or diagnosis (Kruskal–Wallis *p* = 0.850), although survivors of brain tumors reported the highest median impairment. This matters because CCSs often experience multiple late effects [17], and their burden of chronic conditions increases with age [3,5]. Our findings align with the international literature linking late effects, life after brain tumors, or stem cell transplantations with a poorer quality of life [18], as well as with qualitative reports on the cumulative, often invisible burden of several late effects on daily life [19]. It should be noted, however, that our use of single self-rated items captured subjective daily-life impairment rather than a standardized quality of life.

Formal comparison with examination-based cohorts is not feasible, because population, denominators, ascertainment, and grading differ [3,5]. Qualitatively, self-report captured readily perceived or difficult-to-measure conditions, such as orthopedic (24.8%), hearing (17.7%), and psychological problems (30.4%), while potentially asymptomatic, instrument-detectable metabolic (14.8%), cardiovascular (13.9%), and renal conditions (10.3%) were reported less often. This likely reflects differences in surveillance, awareness, and ascertainment rather than prevalence, highlighting the complementary value of self-report and clinical findings.

These findings support structured, specialized LTFU care to detect late effects early and provide coordinated, risk-adapted care, particularly for CCSs with multiple conditions. Targeted interventions, including pain management, psychological support, endocrine substitution, and rehabilitation, may directly improve daily-life impairment and improve quality of life.

### 4.3. The Information Gap on Late Effects

This high burden coexists with a substantial information gap: although most respondents were aware of late effects, the majority felt insufficiently informed, and a small group first learned about these through the survey. This gap persisted across all age-related subgroups and was greatest among survivors treated at a very young age, who cannot receive such information themselves during treatment and may remain underinformed unless it is revisited later. Similar knowledge gaps have been reported in US cohorts [20,21] or regarding specific topics, such as fertility-related late effects [22,23]; however, to our knowledge, this is the first survey addressing this issue in general among CCSs in Germany. As sufficient information did not increase with time since diagnosis, information should be provided repeatedly throughout follow-up and adapted to developmental stage. Parents can bridge early years, while structured transitions, re-education, and survivorship care plans with personalized late-effect recommendations can ensure knowledge transfer and proactive communication [24].

The information gap was greatest among female than male survivors, but smallest among CCSs attending specialized LTFU centers. This aligns with evidence that attending specialized survivorship care improves knowledge of diagnosis, treatments, and late-effect risks [25,26]. In fragmented care, survivors often manage their health largely by themselves [27,28], underscoring the role of care organization in closing the information gap.

### 4.4. Fragmentation of Current Care

Only about half of CCSs reported current or reinitiated adult follow-up care, indicating substantial discontinuity. The stated reasons, limited information or access and follow-up deemed unnecessary by physicians, suggest structural rather than motivational barriers; few reported avoiding cancer-related reminders. This may be underestimated, as such CCSs may have been less likely to participate in the survey or report a lack of information. Overall, structural fragmentation, rather than survivor willingness, appears to be the main barrier.

Adult follow-up was markedly heterogeneous. Most CCSs attended a community specialist or general practitioner, some remained in pediatric oncology, and only one in four reported access to specialized LTFU care. While adult providers are generally appropriate, they may lack CCS-specific expertise, as CCSs play only a minor role in their daily practice. Current evidence does not favor exclusively center-based care over well-coordinated, guideline-based shared care; here, risk-adapted surveillance, multidisciplinary expertise, and coordination are key. Heterogeneous care is not always inappropriate, as survivors differ in exposure, morbidity, and risk [29]. However, it becomes problematic when responsibilities are unclear and no clinician retains oversight. A designated coordinator could address these gaps, consistent with the S2k guidelines’ emphasis on coordinated multidisciplinary care and structured transitions [9], including early preparation, a written treatment summary, an individual risk-adapted follow-up plan, identification of adult providers, and active information transfer. This is particularly relevant, as more than one third reported follow-up ending at or after transition.

### 4.5. Survivor Preferences: Implications for the Design of LTFU Care

Survivors’ preferences reinforce this need, as nearly 60% preferred specialized, multidisciplinary LTFU care, while satisfaction with their current care was low despite its highly perceived importance. Cost coverage was rated highest, implying that LTFU care should be delivered in a setting where all associated costs are covered by health insurance. At present, reimbursement remains incomplete for some recommended examinations, such as dermatological screening or breast surveillance by MRI or ultrasound after chest irradiation. Specialized centers may facilitate reimbursement by providing supporting expertise and evidence. Survivors also prioritized sufficient consultation time, which may be more challenging to provide in routine community care. Similar priorities emerged in a recent Canadian COM-B analysis, in which time constraints and structural barriers limited LTFU engagement while accessibility and financial support acted as enablers [30], suggesting that these factors shape engagement across healthcare systems.

Psychological support showed a further mismatch between current provision and CCS preferences. This aligns with the increased risk of psychological distress among CCSs [4,6] and the frequent, highly impairing psychological problems reported here. In line with the S2k guidelines [9], routine screening and access to psycho-oncology, psycho-therapy, social services, or neuropsychological assessment should be integrated into LTFU care, ideally within a multidisciplinary setting.

Finally, more than 80% of CCSs were willing to travel up to two hours or more for specialized LTFU care, supporting centralized expertise. However, travel may impose barriers due to time, costs, and inequity. As current travel times and residence were not assessed, the potential effect of this burden remains unclear. Together with the high priority to cost coverage, these findings highlight the need for sustainable, equitable access. Overall, survivors’ priorities align with the German S2k guidelines [9] and international survivorship models [31,32], supporting risk-adapted, multidisciplinary, coordinated LTFU care. Internationally, the organization of LTFU care varies considerably, ranging from specialized survivorship clinics in North America and nationally coordinated networks of late-effect clinics, such as the Dutch LATER consortium, to risk-stratified, partly nurse-led aftercare pathways in the United Kingdom [33]. European initiatives, such as PanCare-FollowUp, aim to harmonize person-centered, guideline-based survivorship care across these heterogeneous systems [7]. Compared with these models, Germany is still in the process of establishing a nationwide network of specialized LTFU services, underscoring the relevance of the survivor-defined priorities reported here.

### 4.6. Strengths and Limitations

Strengths include the sizeable, nationwide, survivor-led sample and combined assessment of late effects, daily-life impact, information, current care, and preferences (Figure S5). Limitations include selection bias due to online recruitment through survivor networks, likely underestimating information gap and limiting estimated on access to and awareness of LTFU care. Second, all data were self-reported and therefore subject to recall bias, misclassification and inability to detect asymptomatic conditions, limiting comparability with examination-based cohorts. Furthermore, denominators varied, and treatment-exposure data were limited. Finally, the cross-sectional design cannot establish the effectiveness or cost–effectiveness of preferred care models, and no control groups were included.

Despite these limitations, the consistency of observed gaps and the alignment of survivors’ priorities with the international evidence [24,25] and the German S2k guidelines [9] support survivor-informed LTFU development and evaluation. Self-report complements, but cannot replace, risk-adapted, exposure-based surveillance of asymptomatic late effects. Involving survivors may improve relevance and acceptability; therefore, survivorship care should be built with and not merely for survivors. The key challenge is establishing sustainable, equitable, and coordinated LTFU care structures across Germany.

## 5. Conclusions

This nationwide, survivor-led survey reveals a high self-perceived late-effect burden, meaningful impairment of daily life, a pronounced information gap, and substantial unmet needs in long-term follow-up care. German childhood cancer survivors strongly favor coordinated, specialized, multidisciplinary LTFU care. These patient-centered findings support structured, risk-adapted long-term follow-up with proactive, comprehensible communication and supportive care, and they support the development of LTFU centers with sustainable financing and integrated psychological support. Self-report surveys and clinical screening are complementary; neither approach alone captures the full burden of surviving childhood cancer. As a survivor-led study, this work demonstrates the value of incorporating lived experience into the design, implementation, and evaluation of survivorship care.

## Figures and Tables

**Table 1:** Characteristics of the study population (N = 339). Data are presented as n (%) unless otherwise indicated. * Multiple treatment modalities possible.

Characteristics	Overall (N = 339)
**Sex, n (%)**	
Female	227 (67.0%)
Male	109 (32.2%)
Diverse	3 (0.9%)
**Age (years)**	
Age at diagnosis, median (Q1–Q3)	10 (4–14)
Age at participation, median (Q1–Q3)	31 (25–38)
Time since diagnosis, median (Q1–Q3)	22 (16.5–30)
**Primary diagnosis, n (%)**	
Leukemia	104 (30.7%)
CNS tumor	62 (18.3%)
Lymphoma	56 (16.5%)
Bone tumor	42 (12.4%)
Soft tissue sarcoma	27 (8.0%)
Neuroblastoma	13 (3.8%)
Germ cell tumor	11 (3.2%)
Renal/Wilms tumor	9 (2.7%)
Hepatic tumor	5 (1.5%)
Carcinoma	4 (1.2%)
Retinoblastoma	3 (0.9%)
Other	3 (0.9%)
Cancer treatment *, n (%)	
Chemotherapy	307 (90.6%)
Surgery	188 (55.5%)
Cranial irradiation	93 (27.4%)
Local irradiation	88 (26.0%)
Total body irradiation	41 (12.1%)
Stem cell transplantation	67 (18.9%)
Other therapy	12 (3.5%)
**Recurrence, n (%)**	
No recurrence	254 (74.9%)
≥1 recurrence	85 (25.1%)

## Data Availability

The original contributions presented in this study are included in the article/Supplementary Material. Further inquiries can be directed to the corresponding author.

## Supplementary Materials

The following supporting information can be downloaded at: https://www.mdpi.com/article/10.3390/cancers18182951/s1, Figure S1. Subjective information status about late effects, shown as the share within each age-related subgroup (%): (a) by age at diagnosis; (b) by diagnosis period; (c) by years since diagnosis. Categories: first learned through the survey that cancer treatment can cause late effects; aware that late effects can occur but insufficiently informed; sufficiently informed about possible late effects. Figure S2. Subjective information status about late effects among CCS, shown as the share within each current adult long-term follow-up (LTFU) care type (%). Categories: first-time hearing about late effects (red); aware but not sufficiently informed (yellow); sufficiently informed (green). Care types with no respondents (n = 0) are displayed as empty rows. Figure S3. Distribution of survivors’ ratings of the importance of long-term follow-up care and satisfaction with current care. The paired analysis included participants with valid responses to both items (n = 172). Figure S4. Survivors willingness to travel beyond their local area for specialized LTFU care. Figure S5. Additional figure providing supplementary analysis on geographical data of participants to support the findings presented in the main manuscript. Material S1. Survey questionnaire administered to participants (English translation (a) and German original version (b)).

## Abbreviations

The following abbreviations are used in this manuscript: CCS—Childhood Cancer Survivor; LTFU—Long-Term Follow-Up; PPI—Patient and Public Involvement; GvHD—Graft-Versus-Host Disease; IGHG—International Late Effects of Childhood Cancer Guideline Harmonization Group; Q1–Q3—Interquartile Range.
