# Supplementary material for: Self-Reported Late Effects, Information Needs, and Preferences for Long-Term Follow-Up Care Among Survivors of Childhood Cancer: A Nationwide Survivor-Led Survey from Germany
Source: Cancers (Basel). 2026 Sep 12;18(18):2951. doi: 10.3390/cancers18182951 (PMC13604324; doi:10.3390/cancers18182951)
Supplement: Supplementary file 1 [file cancers-18-02951-s001.zip › cancers-4534417-supplementary.pdf]

Supplementary material

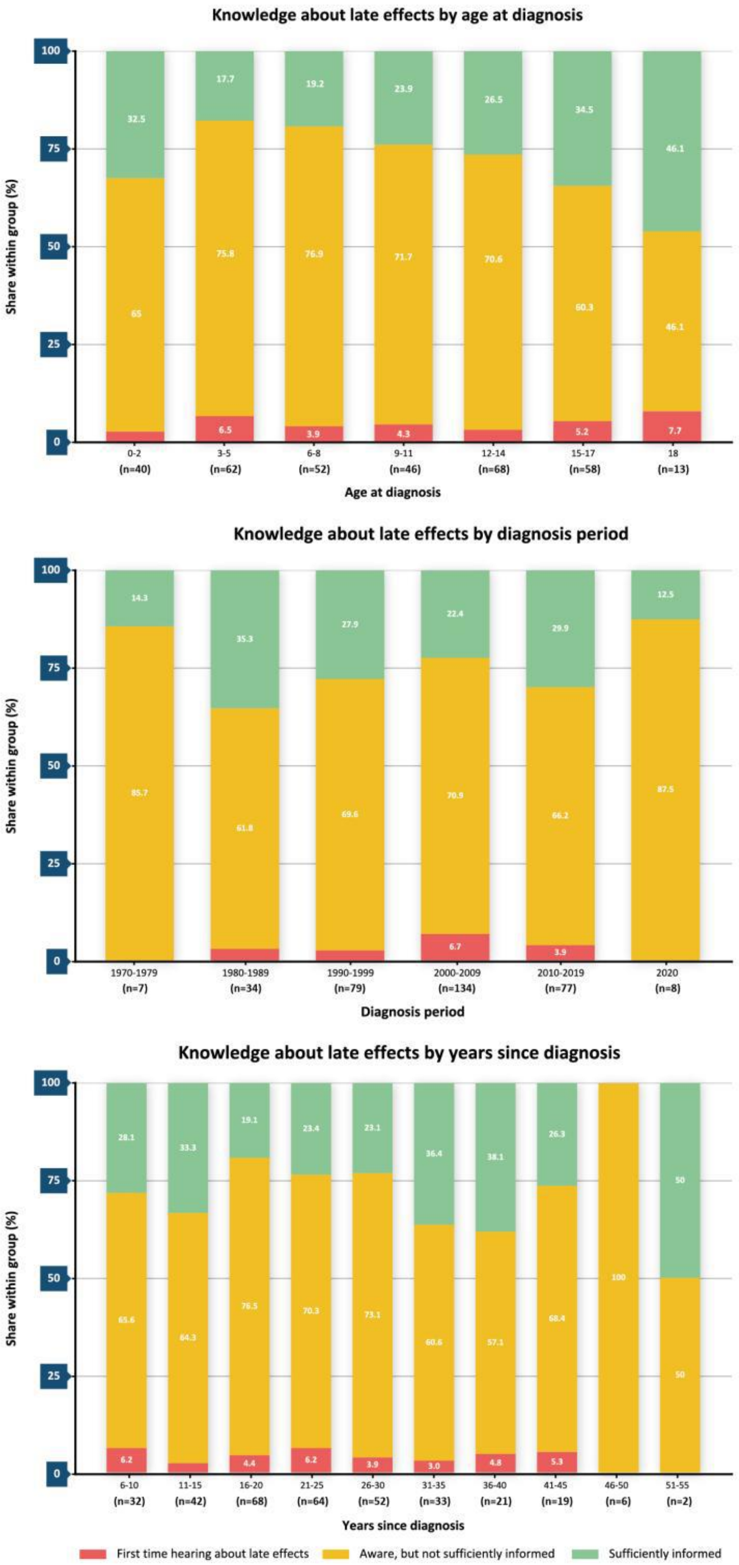

Supplementary Figure S1. Subjective information status about late effects, shown as the share within each age-related subgroup (%): (a) by age at diagnosis; (b) by diagnosis period; (c) by years since diagnosis. Categories: first learned through the survey that cancer treatment can cause late effects; aware that late effects can occur but insufficiently informed; sufficiently informed about possible late effects.

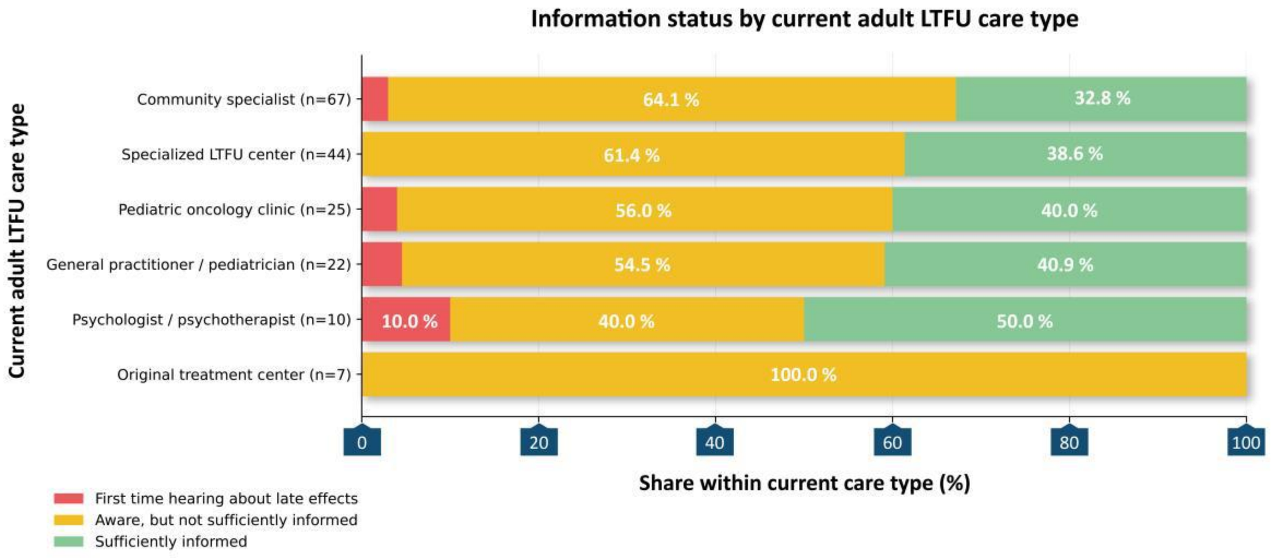

Supplementary Figure S2. Subjective information status about late effects among CCS, shown as the share within each current adult long-term follow-up (LTFU) care type (%). Categories: first-time hearing about late effects (red); aware but not sufficiently informed (yellow); sufficiently informed (green). Care types with no respondents (n = 0) are displayed as empty rows.

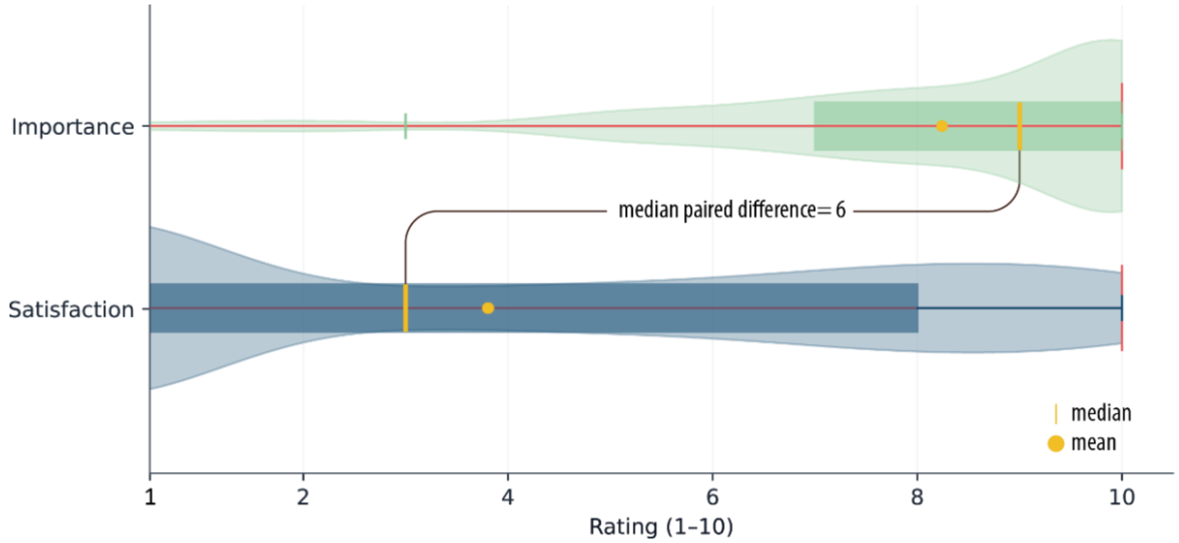

Supplementary Figure S3. Distribution of survivors' ratings of the importance of long-term follow-up care and satisfaction with current care. The paired analysis included participants with valid responses to both items (n = 172).

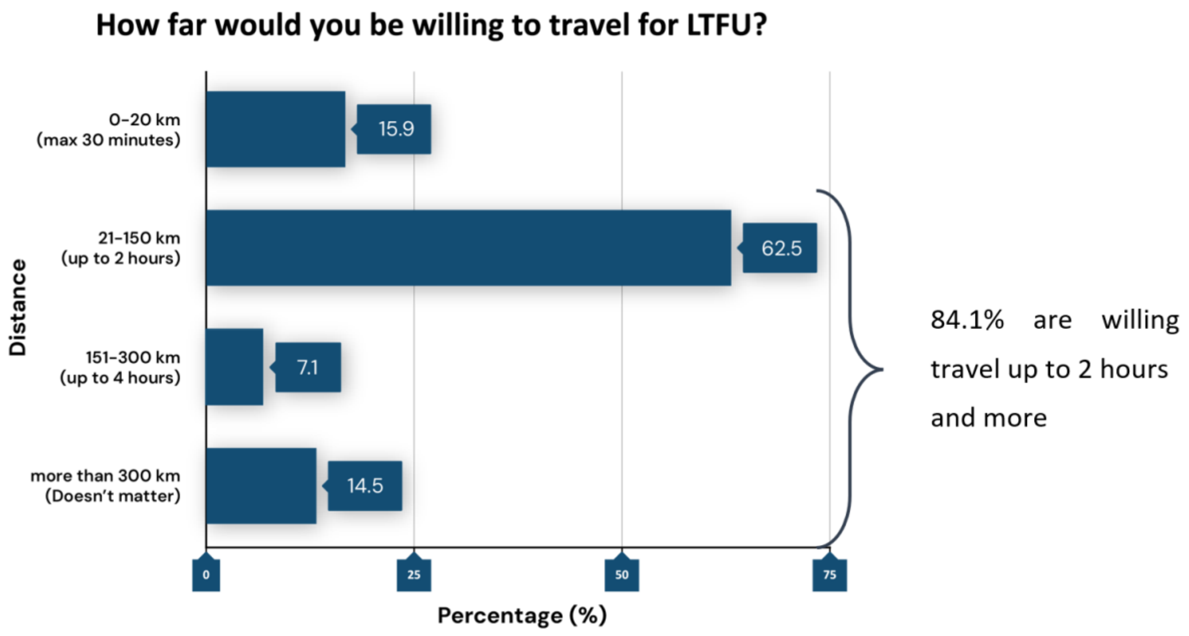

Supplementary Figure S4. Survivors willingness to travel beyond their local area for specialized LTFU care.

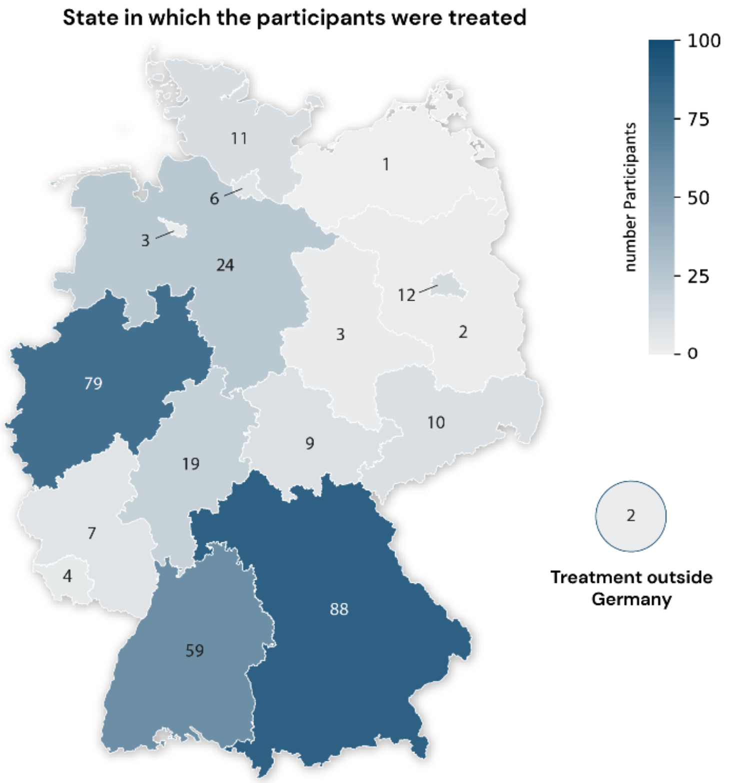

Supplementary Figure S5. Additional figure providing supplementary analysis on geographical data of participants to support the findings presented in the main manuscript.

Supplementary material S1. Survey questionnaire administered to participants (English translation (a) and German original version(b))

S2 a) Survey for Childhood Cancer Survivors

Part 1: Current Follow-up Care

1. Year of birth
2. Year of cancer diagnosis (initial diagnosis)
3. In which federal state was the clinic where you received your treatment located?
    a. Baden-Württemberg
    b. Bavaria
    c. Berlin
    d. Brandenburg
    e. Bremen
    f. Hamburg
    g. Hessen
    h. Mecklenburg-Western Pomerania
    i. Lower Saxony
    j. North Rhine-Westphalia
    k. Outside Germany
4. Sex
5. What type of cancer were you diagnosed with?
    a. Leukemia (e.g. ALL, AML)
    b. Lymphoma (e.g., Hodgkin lymphoma, Burkitt lymphoma)
    c. Central nervous system (CNS) tumor (e.g., medulloblastoma, astrocytoma)
    d. Peripheral nervous system tumor (e.g., neuroblastoma)
    e. Retinoblastoma
    f. Kidney tumor (e.g., Wilms tumor)
    g. Liver tumor (e.g., hepatoblastoma)
    h. Bone tumor (e.g., osteosarcoma, Ewing sarcoma)
    i. Soft tissue sarcoma (e.g., rhabdomyosarcoma)
    j. Germ cell tumor
    k. Carcinoma
    l. Other
6. Have you experienced a relapse?
    a. Yes
    b. No
7. If you experienced a relapse, in which year did it occur?

Part 2: Late Effects

8. Have you been diagnosed with any late effects?
    a. Yes
    b. No
9. How well informed do you feel about late effects?
    a. I feel sufficiently informed about the possible late effects of cancer treatment.

    b. I am aware that cancer treatment can cause late effects, but I do not feel sufficiently informed about them.
    c. This is the first time I have heard about the possibility of late effects resulting from cancer treatment.
10. Which late effects have you been diagnosed with?
    a. Cardiovascular problems (e.g. heart attack, stroke, heart failure)
    b. Problems with hormones (e.g. diabetes, hyperthyroidism, hypothyroidism, growth, hormone deficiency)
    c. Secondary cancer (which?)
    d. Problems with fertility (e.g. absent menstruation, infertility, premature menopause, testosterone deficiency)
    e. Problems with the kidneys (e.g. impaired kidney function)
    f. Metabolic disorders (e.g. dyslipidemia)
    g. Orthopedic problems (e.g. osteonecrosis, problems related to the surgical site)
    h. Visual impairment
    i. Hearing impairment
    j. Fatigue
    k. Neurocognitive problems (e.g. difficulties with concentration, learning difficulties)
    l. Neurological Problems (e.g. spasticity, polyneuropathy)
    m. Chronic pain
    n. Psychological problems
    o. Dental problems
    p. Graft-versus-host disease (GvHD; following hematopoietic stem cell transplantation)
    q. Other
11. How much do you feel limited in your daily life by your late effects?
    a. 1=not at all to 10=extremely
12. Please use the space below to share your experiences with late effects
    a. Free-text response

Part 3: Current Follow-up care

13. Have you received regular follow-up care?
    a. Yes, until I was 18 years old
    b. Yes, until now
    c. Yes, but only starting at a later point of time
    d. Yes, but not anymore
    e. No
14. Why are you currently not receiving follow-up care?
    a. I moved
    b. I do not think it is necessary
    c. I do not want to be reminded of my illness anymore
    d. My doctor told me I do not need any follow-up care
    e. No suitable follow-up care is available
    f. I lack information about available follow-up care or the need for it.
    g. Other: free-text response
15. Where did you receive your follow-up care before your 18th birthday?

   a. I did not receive follow-up care
   b. At a pediatrician’s or general practitioner’s office
   c. At a community-based oncologist or another community-based medical specialist office
   d. At a pediatric oncology follow-up clinic
   e. At the center where I received my cancer treatment
   f. At a specialized long-term follow-up center
   g. At a psychologist’s office
   h. At a naturopath’s, homeopath’s, or osteopath’s practice
   i. Somewhere else: free-text response
16. Where did you receive your follow-up care after your 18th birthday?
   a. I did not receive any follow-up care
   b. At a pediatrician’s or general practitioner’s office
   c. At a community-based oncologist or another community-based medical specialist office
   d. At a pediatric oncology follow-up clinic
   e. At the center where I received my cancer treatment
   f. At a specialized long-term follow-up center
   g. At a psychologist’s office
   h. At a naturopath’s, homeopath’s, or osteopath’s practice
   i. Somewhere else: free-text response
17. Is/was your follow-up care provided at the same location where you received treatment for your childhood cancer disease?
   a. Yes, at the same pediatric oncology department
   b. Yes, at the same hospital, but in a different department
   c. Yes, in the same city, but at a different healthcare facility
   d. No, in a different city
18. How often do you currently receive follow-up care?
   a. More than once a year
   b. Once a year
   c. Every 2 years
   d. Less often than every 2 years
19. Please use the space below to share your experiences with long-term follow-up care:
   a. Free-text response

Part 4: Preferred follow-up care

20. How important is regular follow-up care to you?
   a. 1=not important at all to 10=very important
21. Where would you prefer to receive follow-up care?
   a. At a general practitioner’s office
   b. At a community-based medical specialist’s office (e.g. oncologist)
   c. At the center where I received my cancer treatment
   d. At a specialized regional referral long-term follow-up care center
   e. At a psychologist’s office
   f. At a naturopath’s, homeopath’s, or osteopath’s practice

g. Via telemedicine (online consultation)
h. Somewhere else: free-text response

22. Please rate how important the following aspects of follow-up care are to you (1=not important, 10=very important)
    a. Proximity to my current place of residence
    b. Being seen by the same physician at every follow-up visit
    c. Communication between my follow-up cate team and my (former) treating team
    d. Having sufficient time during follow-up appointments
    e. Being able to have all examinations needed on the same day
    f. Receiving a reminder before each follow-up visit
    g. Being cared for by an internist (adult medicine physician)
    h. Continuing to be cared for by a pediatric care team
    i. Having one dedicated contact person for all my medical late effects
    j. Being able to easily reach my follow-up care team
    k. Receiving timely feedback on examination results and recommendations after the appointment
    l. Having all costs covered by my health insurance
23. How far would you be willing to travel for appropriate follow-up care?
    a. 0-20 km (approximately 30 min)
    b. 21-150 km (approximately 2 hours)
    c. 151-300 km (approximately 3-4 hours)
    d. More than 300 km (distance does not matter)
24. Which method of communication would you prefer for contacting your follow-up care team?
    a. Telephone
    b. E-mail
    c. Online portal/app
    d. Other: free-text response
25. What else would you like to see in your follow-up care? Do you have any suggestions for improving your current follow-up care?
    a. Free-text response

S2 b) <u>Fragebogen für Survivor</u>

Teil 1: Angaben zur aktuellen Nachsorge

1. Geburtsjahr
2. Erkrankungsjahr (Ersterkrankung)
3. In welchem Bundesland befindet sich die Klinik, in der Sie/Du behandelt wurdest?
    a. Baden-Württemberg
    b. Bayern
    c. Berlin
    d. Brandenburg
    e. Bremen
    f. Hamburg
    g. Hessen

h. Mecklenburg-Vorpommern
i. Niedersachsen
j. Nordrhein-Westfalen
k. Außerhalb Deutschlands

4. Geschlecht
5. Welche Krebsart wurde bei dir diagnostiziert?
    a. Leukämie (ALL, AML, …)
    b. Lymphom (Hodgin-Lymphom, Burkitt-Lymphom, …)
    c. ZNS-Tumor (Medulloblastom, Astrozytom, …)
    d. Peripherer Nervenzelltumor (Neuroblastom, …)
    e. Retinoblastom
    f. Nierentumor (Wilmstumor, …)
    g. Lebertumor (Hepatoblastom, …)
    h. Knochentumor (Osteosarkom, Ewing-Sarkom, …)
    i. Weichteilsarkom (Rhabdomyosarkom, …)
    j. Keimzelltumor
    k. Karzinom
    l. Andere
6. Hast du ein Rezidiv erlebt?
    a. Ja
    b. Nein
7. Wenn ein Rezidiv aufgetreten ist, in welchem Jahr war das?

Teil 2: Spätfolgen

8. Gibt es bei dir bereits bekannte Spätfolgen?
    a. Ja
    b. Nein
9. Wie gut fühlst du dich zum Thema Spätfolgen informiert?
    a. Ich fühle mich ausreichend über mögliche Spätfolgen der Therapie informiert.
    b. Ich weiß, dass es Spätfolgen durch die Therapie gibt, fühle mich aber nicht ausreichend informiert.
    c. Ich höre zum ersten Mal von möglichen Spätfolgen durch die Therapie.
10. Welche Spätfolgen sind bei dir bereits diagnostiziert worden?
    a. Probleme mit dem Herzen oder Gefäßsystem (z.B. Herzinfarkt, Schlaganfall, Herzschwäche)
    b. Probleme mit Hormonen (z.B. Diabetes, Schilddrüsenüberfunktion oder Schilddrüsenunterfunktion, Wachstumshormonmangel)
    c. Zweittumor (Welcher?)
    d. Fruchtbarkeitsprobleme (z.B. ausbleibende Menstruation, unerfüllter Kinderwunsch, verfrühte Menopause, Testosteronmangel)
    e. Probleme mit den Nieren (z.B. Einschränkung der Nierenfunktion)
    f. Stoffwechselprobleme (z.B. Fettstoffwechselprobleme)
    g. Orthopädisch (z.B. Knochennekrosen, Probleme im Operationsgebiet)
    h. Sehbeeinträchtigungen
    i. Probleme mit dem Hören

j. Fatigue
k. Neurokognitive Probleme (z.B. Konzentrationsprobleme, Lernschwierigkeiten)
l. Neurologische Probleme (z.B. Spastik, Polyneuropathien)
m. Chronische Schmerzen
n. Psychische Probleme
o. Probleme mit den Zähnen
p. Spender-gegen-Empfänger-Reaktion (GvHD nach z.B. Stammzelltransplantation)
q. Sonstiges:

11. Wie sehr fühlst du dich durch deine Spätfolgen im Alltag eingeschränkt?
    a. 1= gar nicht bis 10= extrem
12. Hier ist Platz für deine Erfahrungen zu Spätfolgen
    a. freitext

Teil 3: aktuelle Nachsorge

13. Warst du bislang regelmäßig bei der Nachsorge?
    a. Ja, bis ich 18 Jahre alt war
    b. Ja, bis jetzt
    c. Ja, aber erst zu einem späteren Zeitpunkt
    d. Ja, aber jetzt nicht mehr
    e. Nein
14. Warum bist du aktuell bei keiner Nachsorge?
    a. Umzug
    b. Ich sehe keine Notwendigkeit
    c. Ich möchte nicht mehr an die Erkrankung erinnert werden
    d. Mein Arzt hat gesagt, ich brauche keine
    e. Kein geeignetes Nachsorgeangebot
    f. Mir fehlen Informationen über ein Angebot oder die Notwendigkeit
    g. Sonstige:
15. Wo fand deine Nachsorge vor dem 18. Geburtstag statt?
    a. Es findet / fand keine Nachsorge statt.
    b. Beim Kinderarzt/der Kinderärztin oder beim Hausarzt/der Hausärztin
    c. Beim niedergelassenen Onkologen/bei anderen niedergelassenen Fachärztin
    d. Als Sprechstunde in der Kinderonkologie
    e. Als Sprechstunde am Zentrum, an dem die Behandlung stattgefunden hat
    f. In einem auf Langzeitnachsorge spezialisiertes Zentrum
    g. Bei einem Psychologen/einer Psychologin
    h. Bei einem Heilpraktiker/einer Heilpraktikerin/einem Homöopathen/Homöopathin/einem Osteopathen/einer Osteopathin
    i. Wo anders:
16. Wo fand deine Nachsorge nach dem 18. Geburtstag statt?
    a. Es findet / fand keine Nachsorge statt.
    b. Beim Kinderarzt/der Kinderärztin oder beim Hausarzt/der Hausärztin
    c. Beim niedergelassenen Onkologen/bei anderen niedergelassenen Fachärztin
    d. Als Sprechstunde in der Kinderonkologie
    e. Als Sprechstunde am Zentrum, an dem die Behandlung stattgefunden hat

f. In einem auf Langzeitnachsorge spezialisiertes Zentrum
g. Bei einem Psychologen/einer Psychologin
h. Bei einem Heilpraktiker/einer Heilpraktikerin/einem Homöopathen/Homöopathin/einem Osteopathen/einer Osteopathin
i. Wo anders:

17. Findet / Fand deine Nachsorge am gleichen Ort statt, an dem du mit deiner kinderonkologischen Erkrankung behandelt wurdest?
    a. Ja in der gleiche Kinderonkologie
    b. Ja im gleichen Krankenhaus, aber in einer anderen Abteilung
    c. Ja in der gleichen Stadt, aber in einer anderen Einrichtung
    d. Nein in einer anderen Stadt
18. Wie oft findet diese Nachsorge zum aktuellen Zeitpunkt statt?
    a. Mehrmals pro Jahr
    b. Einmal pro Jahr
    c. Alle 2 Jahre
    d. Mehr als alle 2 Jahre
19. Hier ist Platz für deine Erfahrungen zur Langzeitnachsorge:
    a. freitext

Teil 4: gewünschte Nachsorge

20. Wie Wichtig ist Dir eine regelmäßige Nachsorge?
    a. 1 = gar nicht bis 10 = Sehr wichtig
21. Wo würdest du am liebsten zur Nachsorge gehen?
    a. Beim Hausarzt
    b. Beim niedergelassenen Facharzt (z.B. Onkologe)
    c. Sprechstunde am Zentrum, an dem die Behandlung stattgefunden hat
    d. Auf Langzeitnachsorge spezialisiertes überregionales Zentrum
    e. Bei einem Psychologen/einer Psychologin
    f. Bei einem Heilpraktiker/einer Heilpraktikerin/einem Homöopathen/Homöopathin/einem Osteopathen/einer Osteopathin
    g. Telemedizinisches Angebot (Online-Sprechstunde)
    h. Wo anders
22. Bitte beurteile, was dir in Bezug auf die Nachsorge unwichtig (1) oder wichtig (10) wäre:
    a. Die Nähe zum aktuellen Wohnort
    b. Dass die Nachsorge immer vom gleichen Arzt durchgeführt wird
    c. Dass sich mein Nachsorgeteam mit meinem (ehemals) behandelnden Team austauscht
    d. Dass sich mein Nachsorgeteam ausreichend Zeit nimmt
    e. Dass alle Untersuchungen an einem Tag stattfinden können
    f. Eine Erinnerung vor jedem Besuch
    g. Von Internisten (Erwachsenenmedizinern) betreut zu werden
    h. Weiter von einem Kinderärzte-Team betreut zu werden
    i. Einen Ansprechpartner für alle meine medizinischen Spätfolgen zu haben
    j. Eine gute Erreichbarkeit des behandelnden Teams

    k. Eine möglichst zeitnahe Rückmeldung zu Untersuchungsergebnissen und Handlungsempfehlungen nach dem Termin
    l. Dass die Krankenkasse alle anfallenden Kosten übernimmt
23. Wie weit würde ich für eine passende Nachsorge fahren?
    a. 0-20 km (ca. 30 Minuten)
    b. 21-150km (ca. 2h)
    c. 151-300 km (ca. 3-4h)
    d. Mehr als 300 km (egal)
24. Welchen Kommunikationsweg würdest du als Kontaktmöglichkeit mit deinem Nachsorgeteam bevorzugen?
    a. Telefon
    b. E-Mail
    c. Online-Portal/App
    d. Sonstige
25. Was würdest du dir für deine Nachsorge noch wünschen? Gibt es aus deiner Sicht bei deiner aktuellen Nachsorge Verbesserungswünsche?
    a. Freitext
